# Description and DNA barcoding of *Tipula* ( *Pterelachisus*) *recondita* sp. n. from the Palaearctic region (Diptera, Tipulidae)

**DOI:** 10.3897/zookeys.192.2364

**Published:** 2012-05-08

**Authors:** Valentin E. Pilipenko, Jukka Salmela, Eero J. Vesterinen

**Affiliations:** 1 Department of Entomology, Faculty of Biology, Moscow State University, 119899 Moscow, Russia; 2 Zoological Museum, Department of Biology, FI-20014 University of Turku, Finland; 3 Section of Ecology, Department of Biology, FI-20014 University of Turku, Finland

**Keywords:** Crane flies, Tipulinae, taxonomy, Finland, Russia, COI

## Abstract

*Tipula (Pterelachisus) recondita* Pilipenko & Salmela, **sp. n.** is described. The new species is collected from two localities: Finland, Kittilä (North boreal ecoregion) and Russia, Primorski kray (Zone of temperate broadleaf and mixed forests). Although variation in the structure of male hypopygium between the Finnish and Russian populations is observed, DNA barcode sequences differ only by three nucleotides (0.2 % K2P distance), supporting presence of one widespread species. K2P minimum distances between the new species and 17 other species of the subgenus range from 5.3 to 15.8 % (mean 8.8 %). The new species is forest-dwelling, known from an old-growth herb-rich forest (Finland) and *Quercus mongolica* forest (Russia). The new species is perhaps closest to *Tipula (Pterelachisus) imitator* Alexander and in lesser extent to *Tipula (Pterelachisus) pauli* Mannheims; the inner gonostylus of both species are illustrated.

## Introduction

Tipulidae (Diptera, Nematocera), or long-palped crane flies, are medium to large sized true flies. Globally, 4269 tipulid species and subspecies are known, of these 1322 occupying the Palaearctic region (Oosterbroek 2011). In general, northwest European tipulid fauna is rather well known (e.g. Salmela 2010, 2011). The majority of the species have large European or Palaearctic ranges, only a few species are known from Fennoscandia or Russian Karelia alone. On the other hand, certain species have disjunct occurrences in northern Fennoscandia and the East Palaearctic region (viz. *Tipula kaisilai* Mannheims, *Tipula subexcisa* Lundström, *Tipula tchukchi* Alexander).

*Tipula* (*Pterelachisus*) Rondani is a northern hemisphere subgenus, totaling over 200 species and subspecies (Oosterbroek 2011). Despite taxonomic monographs covering Russia (former USSR, Savchenko 1964) and Europe (Theowald 1980), the Palaearctic fauna of the subgenus includes several elusive species, known from the type locality or female specimens only. Finnish *Tipula* (*Pterelachisus*) species were reviewed by Salmela (2009) and those of the Central European territory of Russia were listed by Pilipenko (2009). The subgenus *Pterelachisus* is closely allied to *Lunatipula* Edwards and *Savtshenkia* Alexander, but is diagnosable due to the bare squama, grayish coloration, patterned wings and structure of male hypopygium (Theowald 1980). Savchenko (1964), dealing with the fauna of former USSR, recognized 11 species groups and two mixed groups within *Tipula* (*Geotipula*) and *Tipula* (*Oreomyza*). These subgenera were synonymized to *Pterelachisus* by Alexander (1965) and later Theowald (1980) named 18 species groups from the West Palaearctic region. These species groups are mainly based on differences in the structure of male hypopygium (Theowald 1980), but no cladistic analysis or phylogeny of the species groups was provided by Theowald or authors after him.

DNA barcoding is a molecular-based method used in the identification and delimitation of species, having usually considerable congruence with morphology-based identifications (Ward et al. 2006, Hausmann et al. 2011, Park et al. 2011). Furthermore, barcoding has revealed cryptic diversity within a morphospecies (Smith et al. 2006, Huemer and Hebert 2011) or indicated a presence of one species despite morphological variation within studied specimens (Memon et al. 2006). DNA barcoding has its disadvantages (Meier 2008, Skevington et al. 2007, Taylor and Harris 2012), but it may be used as an additional, and apparently very powerful, method in taxonomy (Schlick-Steiner et al. 2010). Despite the wide use of DNA barcodes in the current taxonomy and biodiversity studies, the method has been only rarely used in taxonomic studies of crane flies (Ujvárosi et al. 2009, Ujvárosi and Bálint 2012).

In the present article we provide a description of *Tipula (Pterelachisus) recondita* Pilipenko & Salmelasp. n. collected from Europe (Finland) and Asia (Russian Far East). Both sexes of the new species are richly illustrated. In addition, mtDNA sequences (COI) were used to assess (i) the conspecific status of disjunct Finnish and Russian populations and (ii) genetic divergence between the new species and 17 consubgeneric species.

## Material and methods

Total DNA of *Tipula (Pterelachisus) recondita* Pilipenko & Salmela sp. n. specimens was extracted using a modified non-destructive salt extraction method (Aljanabi and Martinez 1997, Gilbert et al. 2007). Whole holotype (JES-20110034) and one paratype (JES-20110035) adult specimens and one leg from a paratype (JES-20110036) were placed on 250 μl 96-plate wells. Ethanol-stored samples were briefly dried at 60 °C. First 118 μl of sterile salt homogenizing buffer (0.4 M NaCl, 10 mM Tris-HCl pH 8.0, 2 Mm EDTA pH 8.0 and 2% SDS) containing 8 μl of 20 mg/ml proteinase K (400 μg/ml final concentration) was added into each well. The samples were incubated overnight in the buffer at 55–65 °C. After the incubation, the intact samples were removed from the buffer and placed into 99.5% ethanol to stop further digestion. Type specimens JES-20110034 and JES-20110035 were finally preserved in 70 % ethanol. Then 80 μl of 6 M NaCl (NaCl saturated H_2_O, pH 8) was added to each well. Samples were vortexed for 1 min at maximum speed, and centrifuged for 20 minutes at 4000 rpm. Thereafter 100 μl of supernatant was transferred to wells on a new plate. An equal volume (100 μl) of isopropanol was added to each sample and the plate was briefly vortexed. Then the plate was placed into freezer (-20 °C) for 1 hour. After freezing, the samples were centrifuged for 20 minutes at 4000 rpm. The supernatant was discarded and the pellet was washed by adding 150 μl of ice-cold 70% ethanol and centrifuging for 20 min at 4000 rpm. The ethanol was then carefully pipetted out and the pellet was dried for overnight at room temperature. The next day, DNA pellet was dissolved in 50 μl of previously warmed ultrapure water.

The DNA barcode region (*cythocrome oxidase subunit I*) was amplified and sequenced from all specimens using universal primers LCO1490: 5’-GGGTCAACAAATCATAAAGATATTGG-3’ and HCO2198: 5’-TAAACTTCAGGGTGACCAAAAAATCA-3’ (Folmer et al. 1994). All PCR reactions were performed in a 20 μl volume containing 1 μl of DNA extract, 12.5 µl ddH_2_O, 2.0 µl 10x buffer, 2.0 µl MgCl_2_, 1.0 µl primer1 (LCO), 1.0 µl Primer 2 (HCO), 0.4 µl dNTPs, and 0.1 µl AmpliTaq Gold polymerase. The cycling profile was 95°C for 5 min, 40 cycles of 94 °C for 30 sec, 50 °C for 30 sec, 72°C for 1 min 30 sec and a final extension period of 72 °C for 10 min. Sterile water samples were used as controls in each PCR batch. All of the controls were negative. Successful PCR products were purified and sequenced by Macrogen Incorporated (South Korea).

For other species (totaling 17 species and 26 specimens, Table 1) DNA barcodes were obtained at the Canadian Centre for DNA Barcoding. Legs or 2–3 abdominal segments of the specimens were placed in 96% ethanol in a 96-well lysis microplate and dispatched to the Biodiversity Institute of Ontario where DNA was extracted and sequenced using standard protocols and primers (deWaard et al. 2008). Resultant sequence data were placed into a project (HOLPT) on BOLD (http://www.boldsystems.org, Ratnasingham and Hebert 2007). These sequence records are now publically available on both BOLD and on GenBank.

**Table 1.** *Tipula* (*Pterelachisus*) specimens used in DNA barcoding (COI). Species and associated BOLD Sample ID are according to HOLPT project, available in http://www.boldsystems.org/. Co-ordinates are given in WGS84 decimal format.

**Sample ID, species**	**Year**	**Country**	**Locality**	**N**	**E**
JES-20110456|Tipula_cinereocincta	2005	Finland	Heinävesi	62.419	28.596
JES-20120024|Tipula_cinereocincta	2007	Finland	Savonranta	62.251	28.877
JES-20120065|Tipula_angulata	2006	Canada	Ontario	45.483	-76.081
JES-20120064|Tipula_entomophthorae	2003	Canada	Manitoba	54.9	-101.43
JES-20120004|Tipula_jutlandica	2008	Finland	Parikkala	61.565	29.559
JES-20110501|Tipula_laetibasis	2002	Finland	Tuupovaara	62.442	30.606
JES-20110497|Tipula_luridorostris	2006	Finland	Taivalkoski	65.785	28.321
JES-20120011|Tipula_mats._pseudohortensis	2007	Finland	Inkoo	60.018	23.822
JES-20110475|Tipula_mats._pseudohortensis	2007	Finland	Siuntio	60.213	24.135
JES-20110092|Tipula_mutila	2009	Finland	Enontekiö	68.639	22.552
JES-20110204|Tipula_mutila	2008	Finland	Kiuruvesi	63.52	26.69
JES-20120095|Tipula_mutila	2007	Finland	Kittilä	68.33	24.64
JES-20120014|Tipula_octomaculata	2008	Finland	Lieksa	63.217	30.218
JES-20120031|Tipula_octomaculata	2006	Finland	Taivalkoski	65.693	28.32
JES-20110494|Tipula_pauli	2007	Russia	Primorski kray	47.94	137.72
JES-20110495|Tipula_pauli	1995	Russia	Moscow region	56.02	37.11
JES-20110502|Tipula_pseudovariipennis	2006	Latvia	Tukums	56.998	23.003
JES-20110035|Tipula_recondita_sp._n	2009	Finland	Kittilä	67.634	25.416
JES-20110034|Tipula_recondita_sp._n	2009	Finland	Kittilä	67.634	25.416
JES-20110036|Tipula_recondita_sp._n	2006	Russia	Primorski kray	43.125	131.4
JES-20120038|Tipula_stenostyla	2009	Finland	Kittilä	67.634	25.416
JES-20110408|Tipula_submarmorata	2009	Finland	Jyväskylä	62.236	25.679
JES-20120041|Tipula_truncorum	2010	Finland	Enontekiö	69.183	21.521
JES-20110345|Tipula_varipennis	2007	Finland	Ranua	66.017	26.852
JES-20120032|Tipula_varipennis	2005	Finland	Jyväskylä	62.213	25.793
JES-20110401|Tipula_varipennis	2009	Finland	Jyväskylä	62.236	25.679
JES-20110222|Tipula_wahlgreni	2008	Finland	Kiuruvesi	63.52	26.69
JES-20110450|Tipula_winthemi	2009	Finland	Lammi	61.091	25.002
JES-20120026|Tipula_winthemi	2008	Finland	Virolahti	60.465	27.426

**Figure 1.**
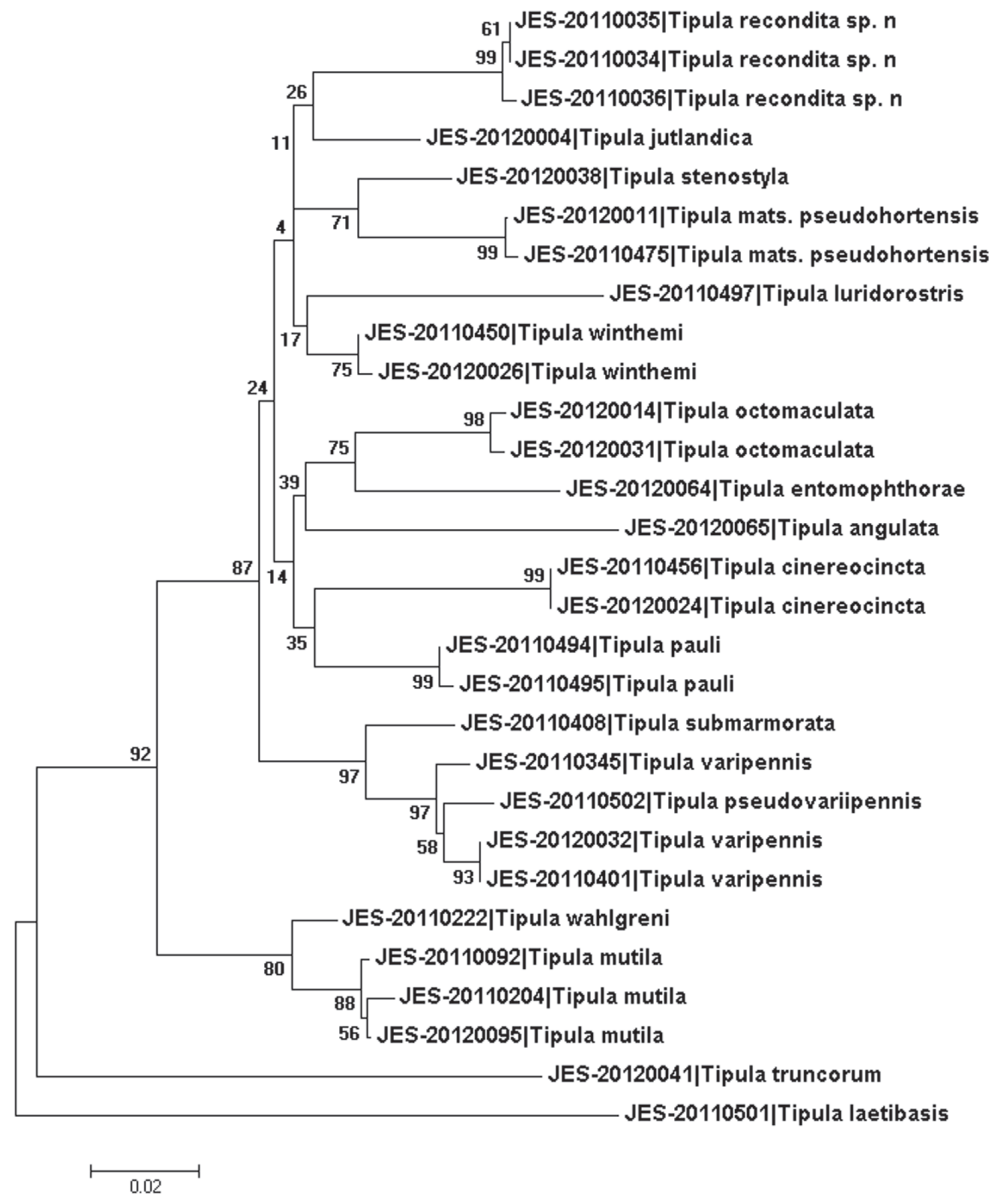
Maximum Likelihood tree based on COI sequences (mtDNA) of 17 *Tipula* (*Pterelachisus*) species. Numerical values denote to Bootstrap values after 1000 replications. In the tree Bootstrap value 26 refers to the clade including *Tipula recondita* sp. n. and *Tipula jutlandica* and value 11 refers to the clade including *Tipula recondita* sp. n., *Tipula jutlandica*, *Tipula stenostyla* and *Tipula matsumuriana psedohortensis*. Scale bar: nucleotide substitutions per site.

In order to assess the COI divergence between the new species and 17 Holarctic *Tipula* (*Pterelachisus*) species, we calculated Kimura two-parameter (K2P) (Kimura 1980) distances between all sequenced specimens. Based on K2P distances we also produced Neighbor-Joining (NJ) tree to visualize similarity of the *Pterelachisus* species. We also inferred relatedness of the species with character based Maximum Likelihood (ML) method (GTR + gamma as evolutionary model, 1000 Bootstrapping replicates). However, because the NJ and ML trees were practically identical, only ML tree is presented (Fig. 1). K2P distances, NJ and ML were produced by using MEGA5 program (Tamura et al. 2011). Because one gene is far too little for reasonable phylogenetic analysis (Gatesy et al. 2007), the ML tree presented here do not reliably illustrate evolutionary relationships among the sequenced taxa.

The morphological terminology used here mainly follows Alexander and Byers (1981). Terminology of some special parts of male genitalia was taken from Frommer (1963). If not otherwise stated, measurements are given in μm. The following acronyms for museums and collections are used in the text: ZMKU – Zoological Museum of National Museum of Natural History, National Academy of Science of Ukraine, Kiev, Ukraine; NCBN – Netherlands Centre for Biodiversity Naturalis, Leiden, the Netherlands; ZMUM – Zoological Museum, Moscow State University, Moscow, Russia; ZMUT – Zoological Museum, University of Turku, Turku, Finland; ZISP – Zoological Institute Russian Academy of Sciences, St. Petersburg, Russia; VPM – Private collection of Valentin Pilipenko, Moscow, Russia.

Specimens were studied with a Zoom Stereo Microscope. Photographs were taken with a Canon PowerShot A640 camera and processed using Combine ZP software. All drawings were prepared from photographs.

[Comparative morphological material examined. *Tipula (Pterelachisus) imitator* Alexander: Russia, Shikotan Island, Kray Sveta cape, 25.VII.1965, V. Ermolenko, 1 male (ZMKU); Russia, Iturup Island (Kuril Is), Kurilsk env., 5.VII.1963; Krilov & Krivolutskaya, 1 male (ZISP). *Tipula (Pterelachisus) pauli* Mannheims: Russia, Moscow region, Chashnikovo, 29.V.1995, V. Pilipenko, 1 male (VPM).]

## Taxonomy

### Tipula (Pterelachisus) recondita

Pilipenko & Salmela sp. n.

urn:lsid:zoobank.org:act:CFBAD0A0-AC21-4067-88E4-C15BCA35CC56

http://species-id.net/wiki/Tipula_recondita

#### Material examined.

Holotype: Male, in alcohol (NCBN). “Finland, Lkoc: Kittilä, Iso Mustavaara, old-growth herb-rich forest, 67.6340°N, 25.4160°E, 30.V.–1.VII. 2009, J. Salmela leg.” (white label, printed) “*Tipula (Pterelachisus) recondita* sp. n./ Pilipenko & Salmela 2011/ HOLOTYPE” (white label, printed) “BOLD sample ID JES-20110034” (white label, printed). Both wings are detached. Only one leg is present, other legs are missing. Tip of abdomen is detached and, including separate sperm pump, preserved in a microvial. This microvial is in the same tube as are wings and rest of the specimen. DNA barcode (524 bp) of holotype (coded JES-20110034|FINTI034-11|*Tipula recondita*):

ATGCTTTTATTATAATTTTTTTTATAGTTATACCTATTATAATTGGAGGATTTGGAAATTGATTAGTACCTTTAATATTAGGTGCCCCTGATATAGCCTTTCCTCGAATAAATAATATAAGTTTTTGAATATTACCTCCTTCACTTACTCTTTTATTAGCTAGTAGTATAGTCGAAAACGGTGCGGGGACTGGATGAACCGTTTATCCCCCACTCTCATCTAGAATTGCCCATACAGGAGCTTCAGTTGATTTAGCCATTTTTTCTCTTCATTTAGCTGGAATTTCTTCAATTTTAGGAGCAGTAAATTTTATTACTACAGTAATTAATATACGATCAAGAGGAATTACTTTAGACCGAATACCTTTATTTGTTTGATCGGTAGTAATTACTGCAGTATTATTACTACTCTCTTTACCTGTATTAGCGGGAGCTATTACTATACTTTTAACTGATCGAAATTTAAATACATCATTTTTTGATCCTGCAGGAGGTGGAGATCCAATTCTTTACCAACATTTATTT

#### Paratypes.

Finland, Lkoc: Kittilä, Iso Mustavaara Nature reserve, herb-rich old-growth forest, 67.6340°N, 25.4160°E, 30.V.–1.VII. 2009, Malaise trap, J. Salmela leg., 2 males (ZMUT, in alcohol [BOLD sample ID JES-20110035] and a pinned specimen). DNA barcode (524 bp) of paratype (JES-20110035|FINTI035-11) is identical to the holotype sequence. Russia, Far East, Primorski kray, Kedrovaya Pad’, oak forest (*Quercus mongolica*), 43.1301°N, 131.5041°E, 7.VII. 2006 V. Pilipenko leg., 3 males and 3 females, deposited in ZSIP (BOLD sample ID JES-20110036), ZMUM, VPM. DNA barcode (524 bp) of paratype male (JES-20110036|FINTI036-11) differs from holotype at three positions (212=C, 473=T, and 515=G). In other words, intraspecific K2P distance between Finnish and Russian specimens was 0.2 %.

#### Diagnosis.

Rather small yellowish brown *Tipula* species (body length: 11 mm male, 12.3 mm female; wing length 11–12.6 mm male, 12.5–13.5 mm female). Scape, pedicel and base of 1^st^ flagellomere yellowish, other flagellomeres brown. Caudal margin of male 9^th^ tergite with a median notch, bearing no tooth or other elevated structures. Outer gonostylus narrow, about as long as inner gonostylus, slightly bent sub-basally. Lower beak of inner gonostylus apically rounded, black. Outer basal lobe of inner gonostylus with 3–4 stout black spines.

#### Description.

**Figure 2.**
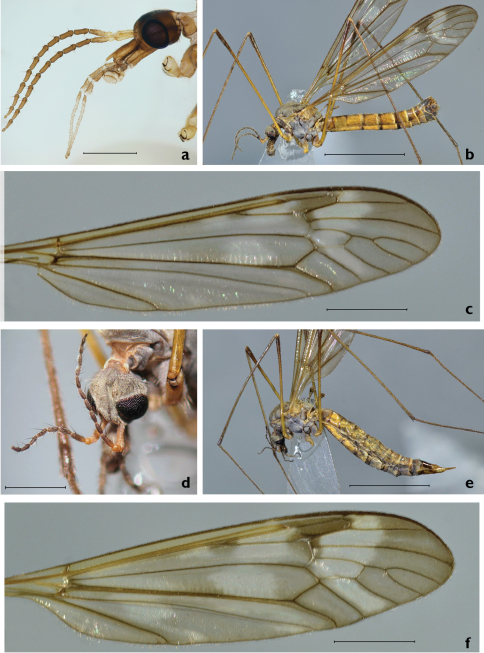
*Tipula (Pterelachisus) recondita* Pilipenko & Salmela, sp. n. **a** Holotype male, head, lateral view (Finland) **b** paratype male, habitus, lateral view (Russia) **c** paratype male, wing (Russia) **d** paratype female, head, dorso-lateral view (Russia) **e** paratype female, habitus, lateral view (Russia) **f** paratype female, wing (Russia). Scale bars: **a, d** 1 mm; **c, f** 2.5 mm; **b, e** 5 mm.

**Figure 3.**
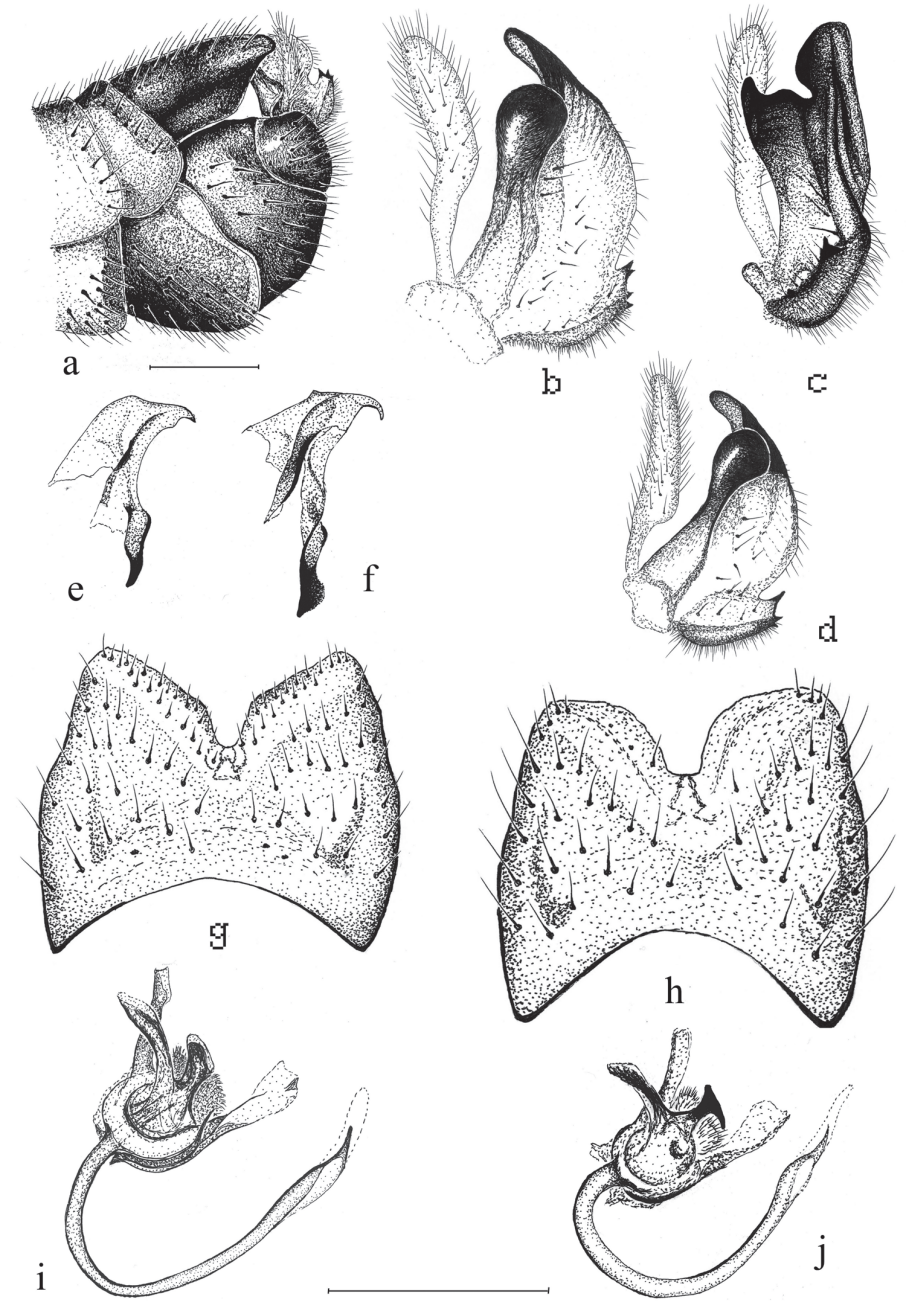
*Tipula (Pterelachisus) recondita* Pilipenko & Salmela, sp. n., paratype males **a** hypopygium, lateral view (Russia) **b** outer and inner gonostylus, lateral view (Finland) **c** outer and inner gonostylus, posterior view (Finland) **d** outer and inner gonostylus, lateral view (Russia) **e** aedeagal guide, lateral view (Finland) **f** aedeagal guide, lateral view (Russia) **g** 9^th^ tergite, dorsal view (Finland) **h** 9^th^ tergite, dorsal view (Russia) **i** sperm pump and aedeagus, ventro-lateral view (Finland) **j** sperm pump and aedeagus, ventro-lateral view (Russia). Scale bars: 0.5 mm.

Male. Head gray pruinose, sparsely covered with dark hairs. Base of rostrum gray pruinose, otherwise dark brown, shining. Nasus distinct, tip with light bristles (Fig. 2a). Palpi brownish. Lengths of palpal segments (n=2): p1 128-147, p2 307-309, p3 317-365, p4 309-333 and p5 1207-1359. Scape, pedicel and base of 1^st^ flagellomere yellowish, other flagellomeres brown. Scape cylindrical (length 442–466, width 119–120, n=2). Pedicel globular (length 132–134, width 134–135, n=2). Flagellar segments cylindrical, covered with silvery, erect and thick pubescence. Verticils black, shorter than respective segments (Fig. 2a). Lengths of flagellomeres (n=2): f1 371–398, f2 312–314, f3 298–316, f4 289–326, f5 297–324, f6 296–325, f7 291, f8 270–289, f9 257–261, f10 227–230 and f11 100. Thorax. General coloration dark brown, with gray pruinosity (Fig. 2b). Pronotum with light hairs. Prescutum with four longitudinal brown bands; lateral bands short, median bands distinctly separated. Anepisternum, katepisternum and anepimeron with dense, gray pruinosity. Scutum, scutellum, laterotergite and mediotergite unicolorous, dark brown. Coxae brown, with light hairs. Trochanters yellowish, with light hairs. Proximal part (ca. two thirds) of femora yellowish, turning dark brown toward tips. Tibiae and tarsi dark brown, spur formula 1:2:2. Tarsal claws smooth. Legs covered with dark brown – black bristles. Stem of halter yellowish, knobs infuscated. Wings with marmorate pattern, length (n= 5) 11.9 mm (11–12.6 mm), venation as in Fig. 2c. R_1+2_ is variable, reach or not reach Costa. Wing cells c and sc yellowish, other cells brown tinged (see Figs. 2b, 2c). Pterostigma distinct. Abdomen yellowish brown, with a narrow dorsal stripe (Fig. 2b). Hypopygium (Fig. 3a) dark brown. Caudal margin of 9^th^ tergite with a median notch, bearing no tooth or other elevated structures (Figs. 3g–h). Caudal margin of 9^th^ tergite oblique (Finnish specimens) or almost horizontal, truncated (Russian specimens) (Figs. 3g–h). Outer gonostylus narrow, about as long as inner gonostylus, slightly bent sub-basally (Figs. 3b, d). Lower beak of inner gonostylus apically rounded, black. Beak of inner gonostylus rather narrow and elongated in lateral view (Figs. 3b, d), tip roundish and proximal margin oblique, notched in posterior view (Fig. 3c). Outer basal lobe of inner gonostylus with 3–4 stout black spines. Aedeagal guide as in Figs. 3e–f. Sperm pump hairy between posterior immovable apodemes, apex of aedeagus pointed (Figs. 3i–j).

**Figure 4.**
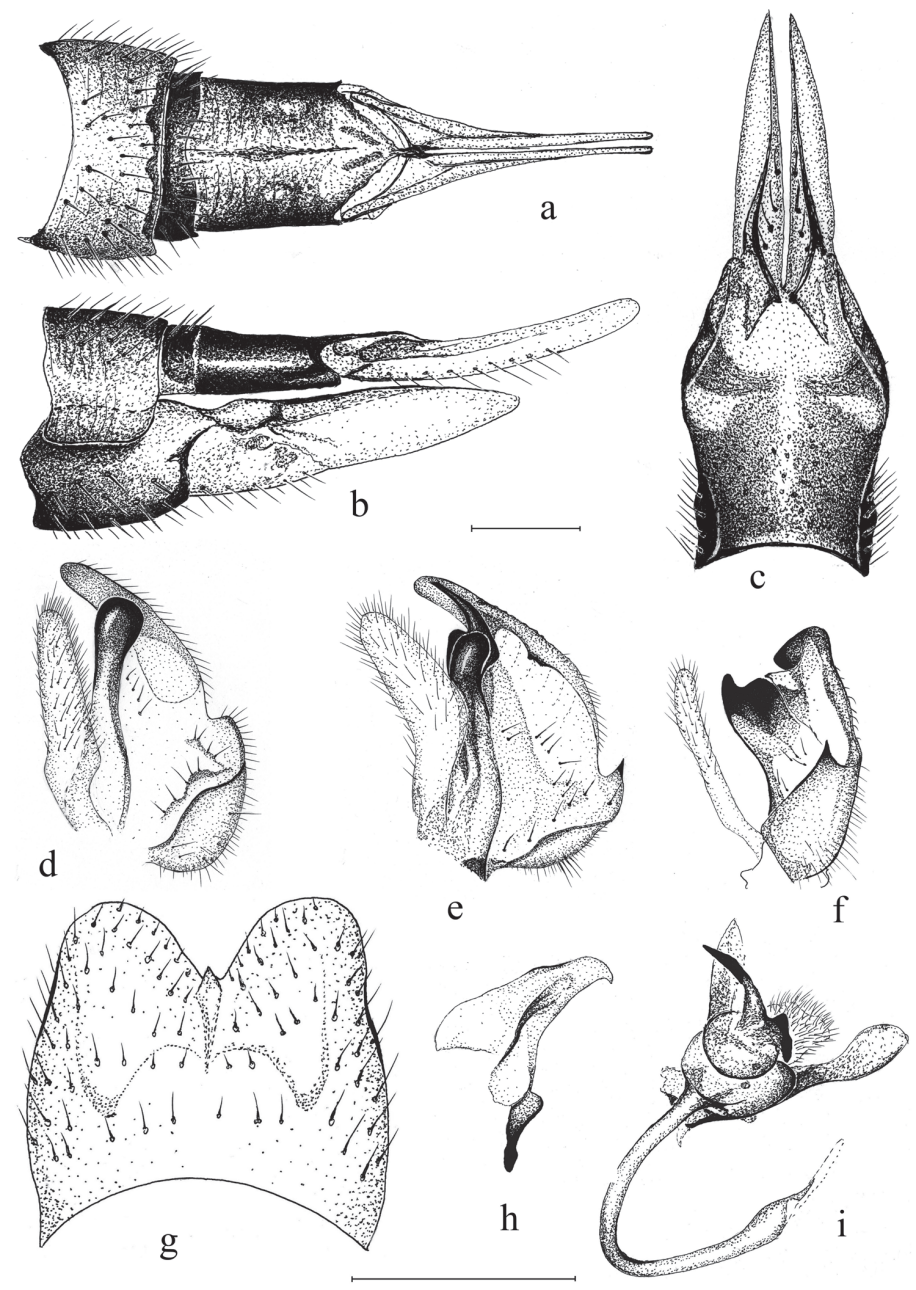
*Tipula (Pterelachisus) recondita* Pilipenko & Salmela sp. n., paratype female (Russia) **a–c** *Tipula (Pterelachisus) pauli* Mannheims **d** and *Tipula (Pterelachisus) imitator* Alexander (**e, f, g, h, i**). **a** female terminal abdominal segments and cerci, dorsal view **b** female terminal abdominal segments, cerci and hypovalva, lateral view **c** female hypovalva and 8^th^ sternite, dorsal view **d–e** male inner gonostylus, lateral view **f** outer and inner gonostylus, posterior view **g** 9^th^ tergite, dorsal view **h** aedeagal guide, lateral view **i** sperm pump and aedeagus, ventro-lateral view. Scale bars: 0.5 mm.

Female. Wing length (n=3) 12.8 mm (12.5–13.5 mm), body length (n=3) 12.3 mm (12–13 mm). Generally similar to male (Fig. 2e). Antenna short (2.4 mm), not extending to wing base (Fig. 2d). The wing’s marmorate pattern more intensive than in male (Fig. 2f). Ovipositor (Figs. 4a, b) elongate, similar to that of most other tipulines; 8^th^ tergite dark brown, 9th tergite narrow dull dark brown, 10^th^ tergite shining chestnut brown. 8^th^ sternite dull dark brown anteriorly, grading to shining yellow posteriorly. Cerci narrow, yellow, slightly longer than 10^th^ tergite. Hypogynial valves yellow, reaching mid-length of cerci, relatively wide, gradually narrowing (Fig. 4c).

#### Etymology.

The species epithet is from reconditus (Latin, adjective) meaning hidden, concealed. This word refers to the rarity and apparent low detectability of the new species, so far known only from two sites in the Palaearctic region.

#### Distribution and ecology.

*Tipula (Pterelachisus) recondita* Pilipenko & Salmela, sp. n. is known from North Europe (Finland) and Asia, Russian Far East. The Finnish collecting site in Kittilä, Iso Mustavaara, is a state-owned Nature Reserve (Lehtojensuojelualue), included in the Natura2000 network of conservation areas. It is part of the biogeographical province of Lkoc (*Lapponia kemensis pars occidentalis*) and lies in the North boreal vegetation zone. The collecting site is an old-growth mixed forest, dominated by birch (*Betula pubescens*), goat willow (*Salix caprea*) and Norway spruce (*Picea abies*), with scattered aspen (*Populus tremula*) trees. Lower vegetation is characterized by herbs and shrubs such as *Calypso bulbosa*, *Daphne mezereum*, *Actaea erythrocarpa*, *Ribes spicatum*, *Filipendula ulmaria* and *Geranium sylvaticum*. Decaying trees, especially goat willow and birch, are abundant in the site. The Russian collecting site is located in the Kedrovaya Pad’ Nature Reserve, within the temperate broadleaf and mixed forest zone, in an oak forest (*Quercus mongolica*) growing on limestone outcrops on the southern slope of a mountain range. Lower vegetation is characterized by *Lespedeza bicolor*, *Spodiopogon sibirieus*, *Astra ageratoides*, *Carex siderosticta*, *Artemisia keiskeana*, *Lathyrus davidii* and *Calamagrostis brachytricha*.

#### Discussion.

*Tipula (Pterelachisus) recondita* Pilipenko & Salmela, sp. n. is rather easily distinguished from other Holarctic *Tipula* (*Pterelachisus*) species. The new species is distinctive in characters of the male hypopygium, especially that of the 9^th^ tergite. There are several *Tipula* (*Pterelachsus*) species with a U-shaped median notch or an emargination in the caudal margin of the tergite, but usually having a tooth or other elevated structures at the mid-point (e.g. *Tipula (Pterelachisus) angulata* Loew [Alexander 1919, p. 984, Salmela and Autio 2007, p. 55], *Tipula (Pterelachisus) varipennis* Meigen [Savchenko 1964, p. 56], *Tipula (Pterelachisus) imitator* Alexander [Alexander 1953, Plate 1], *Tipula (Pterelachisus) resupina* Alexander [Alexander 1935, Plate 2]); the new species is peculiar having no such structures in the 9^th^ tergite.

Morphologically the new species is perhaps the closest to two Palaearctic species, namely *Tipula (Pterelachisus) imitator* and *Tipula (Pterelachisus) pauli*. The former species has a median notch in 9^th^ tergite, but also a distinct tooth at the midpoint (Fig. 4g); the outer basal lobe of inner gonostylus bears one conspicuous black spine, not 3–4 smaller ones (Fig. 4e). For other differences, see Figures 4f, h, i. *Tipula pauli* also has a median notch in 9^th^ tergite and a small but discernible tooth in the midpoint; the lower beak of inner gonostylus is roundish and black, but the outer basal lobe bears no stout, black spines (Fig. 4d). *Tipula (Pterelachisus) imitator* is known from Japan and Kuril Islands and *Tipula (Pterelachisus) pauli* from Europe, Altay and Russian Far East (Oosterbroek 2012, V. Pilipenko pers. obs.).

Based on COI divergence, the new species is apparently rather isolated from the members of the subgenus *Pterelachisus* (Fig. 1). Among the other species vs. the new species, interspecific distances varied from 5.3 % (*Tipula winthemi* Lackschewitz) to 16.1 % (*Tipula laetibasis* Alexander). Mean of the minimum interspecific distances was 8.8 %. According to K2P divergence, the new species is closest to *Tipula winthemi* (5.3 %), *Tipula jutlandica* Nielsen (5.5 %), *Tipula stenostyla* Savchenko (6.6 %) and *Tipula pauli* (6.8 %); distances between the other species range from 7.4 to 16.1 %. In other words, no very close relatives were present in the pair-wise comparisons of COI sequences. For example, much shorter interspecific K2P distances were found between *Tipula varipennis*/*Tipula pseudovariipennis* (1.5 %), *Tipula mutila*/*Tipula wahlgreni* (2.2 %), *Tipula stenostyla*/*Tipula winthemi* (3.7 %). However, it must be emphasized that *Tipula imitator* was not included in COI analysis, due to the lack of fresh material. Given to the morphological similarity of the new species and *Tipula imitator*, it is likely that their barcoding distances would be similar to those three comparisons given above.

There are some morphological differences (9^th^ tergite, inner gonostylus) between Finnish and Russian specimens, perhaps due to the long distance and lack of gene flow between the populations. These differences, however, are here considered to be intraspecific variation. Very small K2P divergence of COI gene (0.2 %) between Finnish and Russian specimens also substantiates the presence of one widespread, but disjunct, species. In rare cases (see Burns et al. 2007) differences of only one to three nucleotides may be observed between otherwise (for example morphologically and ecologically) distinct species. However, in this case we were able to produce 524 bp of high quality sequence, instead of <400 as in the problematic cases of Burns et al. (2007). Moreover, the known biologies of the Finnish and Russian individuals seem alike. To say more of the COI variation, it would be essential to collect more individuals which is rather difficult, given the rarity of the species.

The new species is most probably a very rare tipulid. Despite the rather long tradition of crane fly taxonomy and faunistics in North Europe, this species has hitherto remained unnoticed. One of the authors (JS) has within 12 years identified some 70 000 crane flies from a Finnish Malaise trapping material consisting of 476 sampling sites and ca. 1670 Malaise trapping months. Thus, despite this relatively large sampling effort, only three specimens from a single locality have been caught. The true range of the species is Palaearctic, whether disjunct or not remains to be seen. In Northwestern Europe the species is likely to occur in the north boreal zone (for further information on boreal ecoregions or vegetation zones, see e.g. Ahti et al. 1968). *Tipula (Pterelachisus) recondita* Pilipenko & Salmela, sp. n. may be confined to old-growth forests, and its rarity is perhaps due to the narrow habitat niche. On the other hand, the new species may be hard to collect using traditional methods. Larval associations of this species are unknown, but some *Tipula* (*Pterelachisus*) species are saproxylic, i.e. dependent on decaying trees. Such species are e.g. *Tipula (Pterelachisus) pseudoirrorata* Goetghebuer and *Tipula (Pterelachisus) stenostyla* Savchenko (Salmela 2009), both of them also encountered in the type locality.

To our regret we were not able to examine the holotype male of *Tipula imitator* (D. Furth, pers. comm.). Description of that species was based on a single male specimen (Alexander 1953). We have however examined other material (two male specimens, see above) that very likely represents *Tipula imitator*. Despite morphological similarity of *Tipula (Pterelachisus) recondita* Pilipenko & Salmela, sp. n. and *Tipula imitator*, we are confident that these are separate taxa, due to the differences in the structure of male hypopygium.

## Supplementary Material

XML Treatment for Tipula (Pterelachisus) recondita
